# Selective Pressure Causes an RNA Virus to Trade Reproductive Fitness for Increased Structural and Thermal Stability of a Viral Enzyme

**DOI:** 10.1371/journal.pgen.1003102

**Published:** 2012-11-29

**Authors:** Moshe Dessau, Daniel Goldhill, Robert L. McBride, Paul E. Turner, Yorgo Modis

**Affiliations:** 1 Department of Molecular Biophysics and Biochemistry, Yale University, New Haven, Connecticut, United States of America; 2 Department of Ecology and Evolutionary Biology, Yale University, New Haven, Connecticut, United States of America; Fred Hutchinson Cancer Research Center, United States of America

## Abstract

The modulation of fitness by single mutational substitutions during environmental change is the most fundamental consequence of natural selection. The antagonistic tradeoffs of pleiotropic mutations that can be selected under changing environments therefore lie at the foundation of evolutionary biology. However, the molecular basis of fitness tradeoffs is rarely determined in terms of how these pleiotropic mutations affect protein structure. Here we use an interdisciplinary approach to study how antagonistic pleiotropy and protein function dictate a fitness tradeoff. We challenged populations of an RNA virus, bacteriophage Φ6, to evolve in a novel temperature environment where heat shock imposed extreme virus mortality. A single amino acid substitution in the viral lysin protein P5 (V207F) favored improved stability, and hence survival of challenged viruses, despite a concomitant tradeoff that decreased viral reproduction. This mutation increased the thermostability of P5. Crystal structures of wild-type, mutant, and ligand-bound P5 reveal the molecular basis of this thermostabilization—the Phe207 side chain fills a hydrophobic cavity that is unoccupied in the wild-type—and identify P5 as a lytic transglycosylase. The mutation did not reduce the enzymatic activity of P5, suggesting that the reproduction tradeoff stems from other factors such as inefficient capsid assembly or disassembly. Our study demonstrates how combining experimental evolution, biochemistry, and structural biology can identify the mechanisms that drive the antagonistic pleiotropic phenotypes of an individual point mutation in the classic evolutionary tug-of-war between survival and reproduction.

## Author Summary

The most fundamental mechanism of natural selection in a changing environment is the modulation of fitness by mutations. It is the tradeoffs offered by these mutations that drive evolution. However, fitness tradeoffs are rarely understood at the molecular level, in terms of how the selected mutations affect protein structure and function. Here, we merge experimental evolution and structural biology to study the fundamental tradeoff between survival and reproduction. We challenged populations of an RNA virus to evolve in a novel temperature environment where heat shock imposed extreme virus mortality. A single mutation in a specific viral protein increased the stability, and hence survival of challenged viruses, despite a concomitant tradeoff that decreased viral reproduction. This mutation increased the thermal stability of the mutant protein. Atomic structures of the wild-type and mutant protein reveal the molecular basis of this stabilization. The mutation did not reduce the enzymatic activity of the protein, suggesting that the reproduction tradeoff stems from other factors, such as inefficient virus assembly or disassembly. Our study uncovers the mechanism that drives the antagonistic effects of an individual point mutation in the classic evolutionary tug-of-war between survival and reproduction.

## Introduction

The ability of a single mutational substitution to modulate fitness across environments is the most important consequence of natural selection under environmental change. Understanding the antagonistic tradeoffs of pleiotropic mutations that promote survival in changing environments is therefore essential for a complete understanding of evolution. However, the molecular basis of fitness tradeoffs caused by pleiotropic mutations is rarely determined in terms of how the mutations affect protein structure. Perhaps the main reason for this intellectual gap is because the fields of structural biology and experimental evolution do not often intersect. Structural studies tend to focus on proximate explanations for protein function stemming directly from structural features, without determining the ultimate consequences of evolved protein changes for fitness across environments at the system level. In contrast, experimental evolution studies have identified that point mutations can be consequential for determining fitness tradeoffs in independently evolving populations facing the same environmental change [1], [2], without elucidating the structural details of how such trade-offs are mediated by functional changes at the protein level. It has been argued that interdisciplinary approaches are necessary for the ‘functional synthesis’ that will advance our understanding of evolutionary biology [3], [4], especially to reveal the mechanistic details of evolutionary novelty and adaptive constraint; however, the necessary mergers between disciplines remain rare [5], [6], [7].

Perhaps the most fundamentally important tradeoff in evolutionary biology is that between survival and reproduction, the cornerstones of evolution by natural selection [8]. It is often assumed that natural selection is driven by genetic changes that promote relative differences in offspring production, or reproduction in close relatives [9]. However, the need for organisms to survive in the face of depleted resources or environmental stressors can be of equal or greater importance for dictating relative differences in fitness. It is evident that the functional properties of proteins could bridge tradeoffs in survival versus reproduction, because the genetic changes underlying a protein may simultaneously affect its stability (survival) as well as operational (reproductive) properties across environments. Thus, adaptive evolution in a changing environment provides a key context for studying how protein changes might mediate the interplay of survival versus reproduction, and for determining which variants are favored to evolve under natural selection. ‘Life-history’ tradeoffs between survival and reproduction have been invoked in the adaptive evolution in a variety of organisms [10], but these examples often hinge on statistical correlations between traits, without attempting to identify the molecular basis of changes in protein function that cause such tradeoffs to arise.

Here we challenged populations of an RNA virus, bacteriophage Φ6 of the cystovirus genus [11], to evolve in a novel temperature environment where heat shock imposed extreme virus mortality. A single amino acid substitution in the viral lysin protein P5 favored improved stability (and hence, survival) of challenged viruses, despite a concomitant tradeoff that decreased viral reproduction. Lysins are lytic proteins that locally degrade the cell wall, either to provide access to the inner bacterial membrane during infection or to release virus progeny by cell lysis [12], [13]. An electron microscopy image reconstruction of the bacteriophage Φ12, a cystovirus related to Φ6, suggests that Φ12 P5 is part of the icosahedral nucleocapsid shell of Φ12 and that P5 may interact with the lipid membrane, which constitutes the outer virus layer [14]. It remains to be confirmed whether P5 has an analogous location in Φ6. Nevertheless, the selected mutation in the Φ6 P5 gene increased the thermostability of P5. Crystal structures of the wildtype and mutant P5 reveal the molecular basis of this thermostabilization and identify P5 as a lytic transglycosylase. We show that loss of P5 enzymatic activity is not the source of the viral reproduction tradeoff, which may instead result from inefficient capsid assembly or disassembly. Our study demonstrates how a combination of experimental evolution and biophysical approaches can be used to discover the mechanistic details that drive antagonistic pleiotropic effects of an individual point mutation in the classic evolutionary tug-of-war between survival and reproduction.

## Results

### Evolution of phage Φ6 under heat shock selects the V207F mutation in the P5 lysin

**Figure 1:**
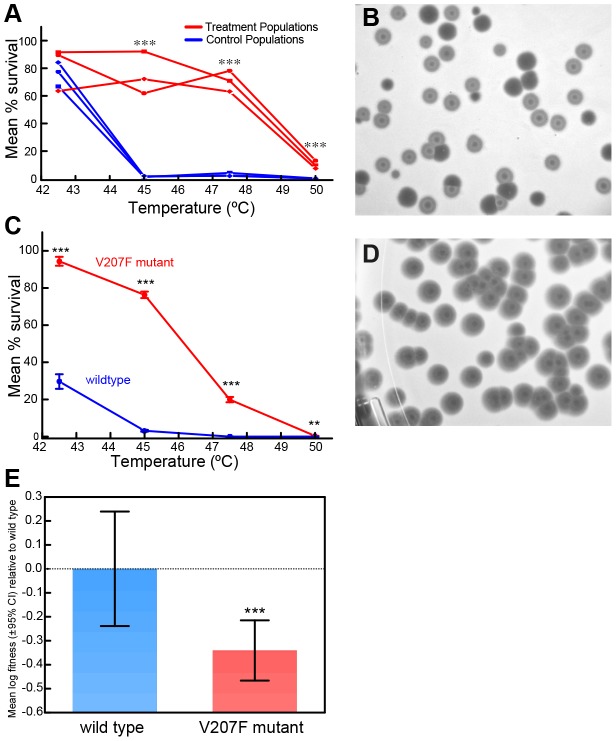
Evolution of Φ6 under thermal pressure. A) Survival of evolved virus lineages as a function of heat shock temperature after a thermal selection with 20 50°C-heat shocks every five generations, or identical passage without heat shock. B) Viruses evolved under heat shock showed a ‘bull's eye’ phenotype in which plaques appeared partially turbid due to residual bacterial growth within the plaque (closed arrows). Normal plaques from wildtype viruses mixed into the sample are labeled with open arrows. C) Survival of a virus genotype with mutation V207F only is greater than the wildtype virus at elevated temperatures. Each point is the mean percent survival (± std. err.) of 3 to 5 survival assays conducted for the strain, and error bars too small to be visualized are omitted for clarity. *** indicates statistical significance at *P*<0.0001, and ** is statistical significance at *P* = 0.004. These results qualitatively agree with those in panel (A) comparing evolved treatment and control populations, with a significant statistical advantage apparent for the V207F mutant at all temperatures tested. D) Bull's eye phenotype of a virus genotype with mutation V207F only. E) The V207F mutation in P5 causes a fitness disadvantage at 25°C relative to wildtype virus; bars indicate means (±95% C.I.). *** indicates statistical significance at *P*≈0.006.

The *P. phaseolicola* host bacteria for phage Φ6 cannot grow and survive at temperatures greater than 30°C. In contrast, virions of wildtype phage Φ6 can withstand exposure to temperatures between 30°C and 40°C, but suffer ‘high mortality’ (subsequent inability to productively infect cells) when subjected to 5 min heat shock at temperatures ranging between 40°C and 50°C [15]. Three populations of wildtype phage Φ6 were evolved independently for 20 days (100 generations) under selection involving 5 min heat shock at 50°C every fifth generation; three control populations were evolved identically, but experienced periodic ‘mock’ heat shocks of 25°C (Figure S1). Subsequently, we conducted repeated (*n* = 3) survival assays at 42.5°C, 45°C, 47.5°C and 50°C for each of the endpoint treatment and control populations. Each treatment population improved in survival in the 50°C selective environment, relative to wildtype phage Φ6 (independent samples t-tests with 5 df, *P*<0.0001). Moreover, the treatment populations were significantly advantaged in survival at temperatures above 45°C relative to the controls, which did not differ in thermotolerance from the ancestor [15] (Figure 1A). These differing thermotolerance ‘reaction norms’ (Figure 1A) clearly demonstrated that evolutionary history affected the evolved ability for treatment versus control populations to withstand elevated temperatures.

To examine whether particular molecular substitutions were associated with the differing thermotolerance phenotypes of the evolved populations (Figure 1A), we obtained the consensus genome sequence for each evolved population. Each evolved lineage differed from the wildtype ancestor by 1 to 6 substitutions; overall we observed 18 substitutions at 9 sites across the 3 genomic segments (Table S1). Interestingly, all three treatment populations showed an identical non-synonymous mutation (G2238T) that was the only molecular change on the small RNA segment, and which was not present in the controls. These data strongly suggested that the mutation was somehow beneficial for adaptation to withstand the 50°C heat shock environment, because it arose spontaneously and fixed in all of the independently evolved treatment (but not control) populations. The G2238T mutation corresponds to a V207F amino-acid substitution in the gene for the lysin protein P5, which locally degrades the cell wall, either to provide access to the inner bacterial membrane during infection or to release virus progeny by cell lysis [12], [13]. Hereafter, we refer to the wildtype protein as P5^wt^ and the mutant protein as P5^V207F^.

Laboratory culture of phage Φ6 on agar always occurs at 25°C, because this incubation temperature allows the *P. phaseolicola* host bacteria to produce a confluent lawn, which supports robust plaque formation of the virus. Intriguingly, we observed that the viruses from the treatment populations showed a novel plaquing phenotype, which is generally referred to as a ‘bull's-eye’ plaque morphology (Figure 1B). Whereas plaques formed by the wildtype virus were clear, the bull's-eye plaques appeared turbid due to residual bacterial growth within the plaque, indicating that mutant viruses were less efficient at killing bacteria at the ordinary growth temperature of 25°C. To confirm that the P5^V207F^ mutation was antagonistically pleiotropic (i.e., caused improved extracellular survival at 50°C but reduced intracellular growth at 25°C), we isolated viruses containing only the P5^V207F^ mutation (Figure 1C). To do so, we conducted a classic genetic cross [1], [2], where an evolved strain bearing only this mutation on the small segment was ‘back-crossed’ with its wildtype Φ6 ancestor, to obtain a hybrid reassortant with a mutated small segment and the ancestral medium and large segments; genotype of the hybrid was confirmed through sequencing. Plaque assays at 25°C showed that the hybrid produced the same bull's-eye plaque morphology that was characteristic of the treatment populations (Figure 1D). In addition, survival assays showed that the thermotolerance reaction norm for the hybrid was qualitatively similar to data observed in the evolved treatment populations (Figure 1A, 1C); at all elevated temperatures survival of the P5^V207F^ mutant significantly exceeded that of the wildtype. Although percent survival of both strains was modest at the extreme 50°C temperature, survival of P5^V207F^ (0.355±0.241 std. dev.) was still greater than the wildtype (0.013±0.007 std. dev.) (t-test with *t* = 3.42, df = 14, *P* = 0.004; Figure 1C). These results indicated that the P5^V207F^ substitution caused both the unique bull's-eye plaque morphology when grown at 25°C, as well as the improved extracellular survival at elevated temperatures.

Because the bull's-eye plaque morphology of the V207F mutant suggested that this genotype less efficiently killed bacteria (relative to the wildtype) at 25°C, we hypothesized that the mutation was deleterious for growth at 25°C. To examine this idea, we conducted paired-growth assays at 25°C, which measured reproduction of the hybrid strain and of the wildtype ancestor under benign conditions, relative to a genetically-marked common competitor virus; (hybrid: n = 28 replicates, wildtype: n = 18 replicates). After adjusting for the cost of the genetic marker on the common competitor, results showed that the mean log reproductive fitness of the mutant relative to the wildtype was −0.341 (±0.324 std. dev.; Figure 1E). Reproductive fitness of the hybrid was significantly less than that of the wildtype based on a two-tailed t-test (with *t* = 2.878, df = 44, *P* = 0.006). Together, we observed that the P5^V207F^ mutation caused a ∼1.5-fold decrease in reproduction at 25°C (Figure 1E), but a ∼27-fold increase in extracellular survival at 50°C (Figure 1C). Results of repeated (n = 5) fitness assays modified to match the treatment conditions (i.e., imposing 50°C heat shock prior to growth on agar at 25°C; Figure S1) also demonstrated the net positive effect of the V207F mutation in the selective environment: mean log fitness of the mutant relative to the wildtype was 3.920 (±0.280 std. err.), which significantly exceeded ancestral log fitness of zero (t-test with *t* = 13.98, df = 5, *P* = 0.0002). We conclude that the P5^V207F^ mutation is an antagonistically pleiotropic allele that produces a survival/reproduction tradeoff across the two portions of the selective environment experienced by the treatment populations; although the mutation is beneficial for extracellular survival under the brief 50°C heat shock, it is deleterious for reproduction occurring at 25°C.

### The V207F mutation enhances the thermostability of P5

**Figure 2:**
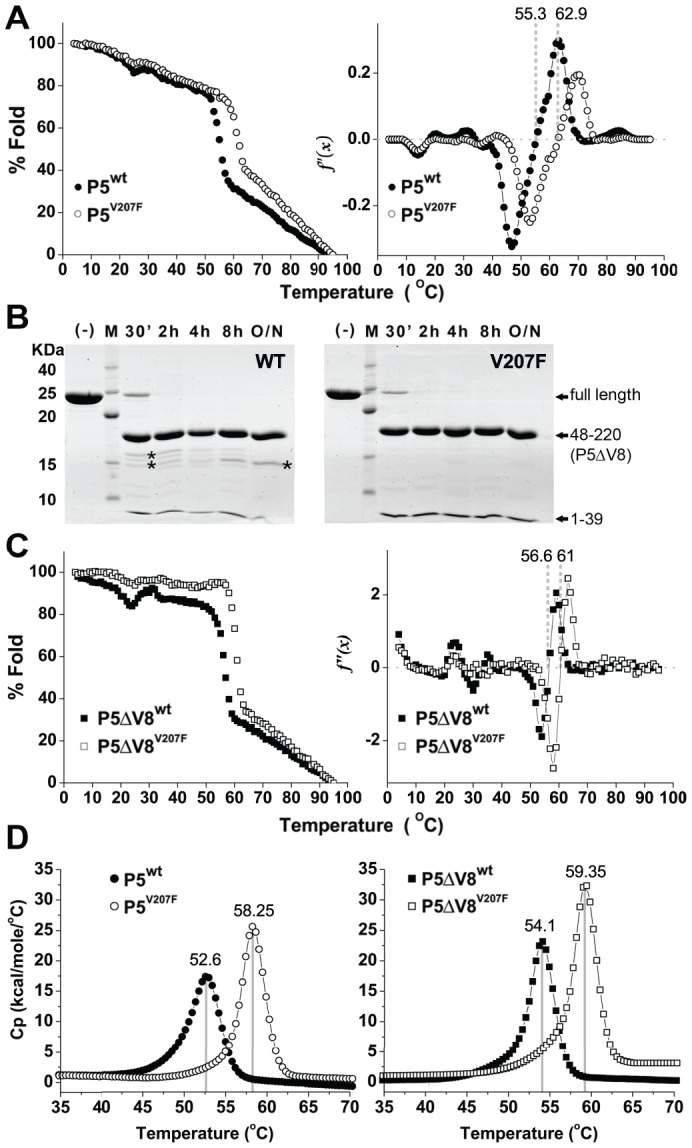
Thermal and proteolytic stabilities of P5^wt^ and P5^V207F^. A) Thermal melting of P5^wt^ and P5^V207F^ measured by circular dichroism (CD) spectrometry at 220 nm. P5^wt^ and P5^V207F^ began to unfold cooperatively at 50–55°C (left panel). Melting temperatures were calculated from the second derivative of the CD melting curves (right panel). The V207F mutation increases the melting temperature of P5 by 7.6°C. B) Limited proteolysis of P5ΔV8^wt^ and P5ΔV8^V207F^ with V8 (Glu-C) protease monitored by SDS-PAGE. Some minor proteolytic products (marked by asterisks) are visible in P5ΔV8^wt^. C) Thermal melting curves of P5ΔV8^wt^ and P5ΔV8^V207F^, with melting temperatures calculated as in (A). D) Differential Scanning Calorimetry (DSC) of P5 and P5ΔV8 proteins. Peaks indicate a 6°C difference between the melting points of wildtype and mutant P5.

The evolved thermotolerance of the viruses carrying the P5^V207F^ mutation could result from an increase in the inherent thermostability of P5, or an increase in the stability of a protein-protein interaction directly or indirectly involving P5. To determine how the V207F mutation in protein P5 induced thermotolerance, we first determined the effect of the mutation on the thermostability of P5. We purified recombinant P5^wt^ and P5^V207F^ from *Escherichia coli*. Thermal melting curves of the two proteins were measured by circular dichroism (CD) spectrometry and differential scanning calorimetry (DSC). P5^wt^ and P5^V207F^ both began to unfold cooperatively as the temperature reached 50°C and 55°C, respectively, as judged from the sharp loss of CD signal from α-helical secondary structure at 220 nm (Figure 2A). Melting temperatures for P5^wt^ and P5^V207F^ calculated from the CD melting curves were 55.3°C and 62.9°C, respectively (Figure 2A). Similarly, the melting temperatures of P5^wt^ and P5^V207F^ determined by DSC were 52.6°C and 58.3°C, respectively (Figure 2). These data indicate that the V207F mutation increases the melting temperature of P5 by between 5.7–7.6°C. Additionally, the heights and areas of the DSC curves were greater for the V207F mutants than for their wildtype counterparts. Together, these data indicate that the higher thermotolerance of the mutant virus is due to increased thermostability of P5^V207F^ relative to P5^wt^. Since melting temperature and DSC peak area depend on the change in entropy and enthalpy, respectively, we conclude that the additional free energy of stabilization from the V207F mutation (Δ*G*) derives from both the entropic term (*T*Δ*S*) and the enthalpy (Δ*H*) in the free energy equation (Δ*G* = Δ*H*−*T*Δ*S*).

### The first 47 residues of P5 are not part of the core fold

The P5 proteins eluted from a size-exclusion column as 40 kDa proteins despite a calculated molecular mass of 25 kDa. We concluded that P5 was either an elongated monomer or a compact monomer with disordered regions that increased the hydrodynamic radius of the protein. Φ6 P5 was previously reported to be a monomer in solution [16]. To determine whether P5 contained regions of disorder or internal flexibility, we subjected P5^wt^ and P5^V207F^ to limited proteolysis with various proteases. Treatment with V8 protease (*Staphylococcus aureus* endoproteinase Glu-C) produced three fragments, which were identified by mass spectroscopy as consisting of residues 1–39, 40–47 and 48–220, respectively (Figure 2B). We will refer to the largest fragment, residues 48–220, as P5ΔV8. Additional proteolytic products were observed for P5^wt^ but not for P5^V207F^ (Figure 2B), revealing a higher overall protease sensitivity of the wildtype protein. P5ΔV8 eluted from a size-exclusion column as would be expected for a globular 19 kDa protein. To investigate the state of residues 1–47 further we calculated the difference CD signal between P5^wt^ and P5ΔV8^wt^ (Figure S2). The minimum at 190 nm indicates that residues 1–47 have essentially no secondary structure. The melting temperatures of P5ΔV8^wt^ and P5ΔV8^V207F^ were similar to the full-length proteins as determined by CD (56.6°C and 61.0°C) and DSC (54.1°C and 59.4°C; Figure 2C, 2D). CD melting curves of the truncated proteins also showed greater stability at temperatures below 50°C than the full-length proteins, suggesting that residues 1–47 are responsible for the observed non-cooperative unfolding of the full-length proteins in this temperature range (Figure 2A, 2C). Together, these data indicate that the first 47 residues of P5 are either disordered in solution or fold separately from the rest of the protein. In the virion, however, residues 1–47 may adopt a stable conformation upon binding other viral components.

### Φ6 P5 adopts a lysozyme superfamily fold

**Figure 3:**
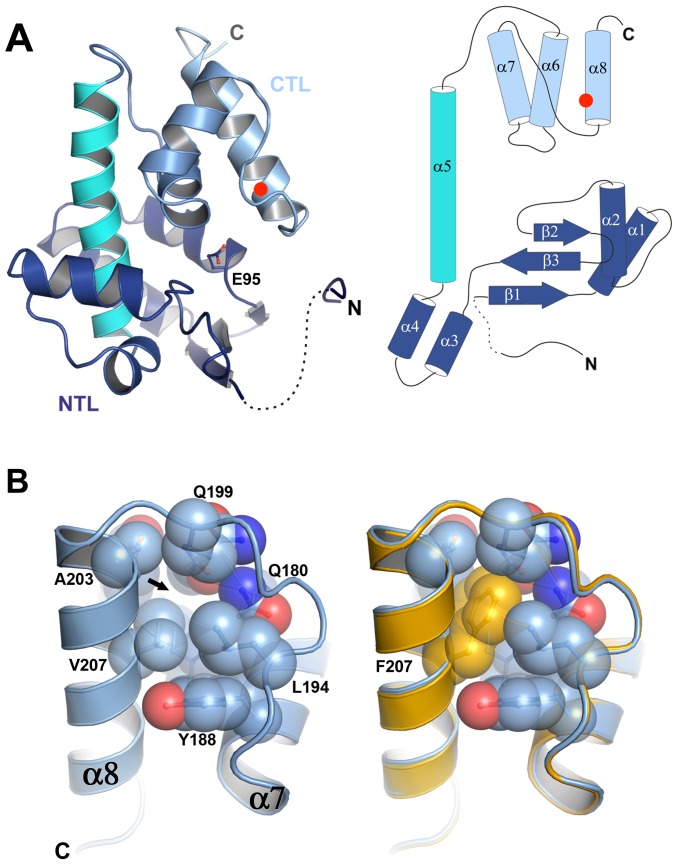
P5 adopts a lysozyme fold and the V207F mutation fills a cavity. A) P5 adopts a lysozyme superfamily fold with an N-terminal lobe (NTL, in dark blue) and a C-terminal lobe (CTL, light blue) connected by a central helix (cyan). The predicted catalytic residue Glu95 lies in the substrate binding cleft between the two lobes. The NTL differs from other lysozyme structures in its overall organization and relative orientation to the CTL. The three N-terminal residues of P5 form an unusual crystal packing interaction. Residues 53–59 are disordered (dashed line). Residue 207 is marked with a red circle. B) Close-up of residue 207. The Phe207 side chain in P5ΔV8^V207F^ (orange) fills a cavity (marked by an arrow) that is unoccupied in P5ΔV8^wt^ (light blue), with no significant changes in the protein backbone (see also Figure S3).

To understand the molecular basis of the thermostabilization of P5 by the V207F mutation we determined the crystal structures of P5ΔV8^wt^ and P5ΔV8^V207F^ at 1.4 Å resolution (we could not obtain crystals of P5^wt^ or P5^V207F^). Crystallographic data collection and refinement statistics are provided in Table S2. P5ΔV8 adopts a lysozyme superfamily fold consisting of an N-terminal lobe (NTL) and a C-terminal lobe (CTL) connected by a central helix (Figure 3A). The two lobes create a substrate binding cleft containing the predicted catalytic residue Glu95 (Figure 3A) [17]. The central helix and the CTL of P5ΔV8 have similar conformations as various other lysozyme structures. However, the NTL in P5 differs from other lysozyme structures in its secondary structure content and relative orientation to the CTL (Figure S3). We note that P5ΔV8 forms an unusual crystal-packing interaction in which three N-terminal residues (residues 48–50) of one of the two subunits in the asymmetric unit insert into a shallow groove in a molecule in the adjacent asymmetric unit (Figure S3F). Given that residues 48–50 make specific crystal contacts it was surprising that residues 53–59 were disordered and only residues 48–52 and 60–220 could be modeled.

### The V207F mutation fills an unoccupied hydrophobic cavity in wild-type P5

The structures of P5ΔV8^wt^ and P5ΔV8^V207F^ are almost identical, with a root mean square deviation (rmsd) of the Cα positions of 0.135 Å (Figure 3B, Figure S3G). Residue 207 is located on helix α8, facing the hydrophobic core of the protein. In P5ΔV8^wt^, V207 and adjacent residues 153, 176, 179–180, 194, 199, and 203–204 create a small (30.28 Å^3^) cavity lined with hydrophobic side chains (Figure 3B, Figure S4). The lack of electron density within the cavity suggests that it is unoccupied. The larger phenylalanine side chain in P5ΔV8^V207F^ neatly fills the cavity, with no significant changes in the protein. The B-factors near residue 207 are similar in both structures (15–16 Å^2^). The only significant side chain shift observed in P5ΔV8^V207F^ is an adjustment in the N179 side chain to accommodate the F207 side chain, resulting in a van der Waals interaction between these two side chains. By filling an unoccupied hydrophobic cavity, the V207F substitution increases the buried hydrophobic surface area in the protein. This provides a likely explanation for the increased thermostability of the mutant, because the hydrophobic surface area that is buried within a folded protein contributes directly to its free energy of stabilization [18], [19]. In support of this explanation, the hydrophobic cavity created by the replacement L99A in T4 lysozyme was large enough to bind benzene, and binding of benzene to the L99A mutant increased the melting temperature of T4 lysozyme by 6.0°C [20].

### The mode of P5 glycan binding suggests a lytic transglycosylase activity

**Figure 4:**
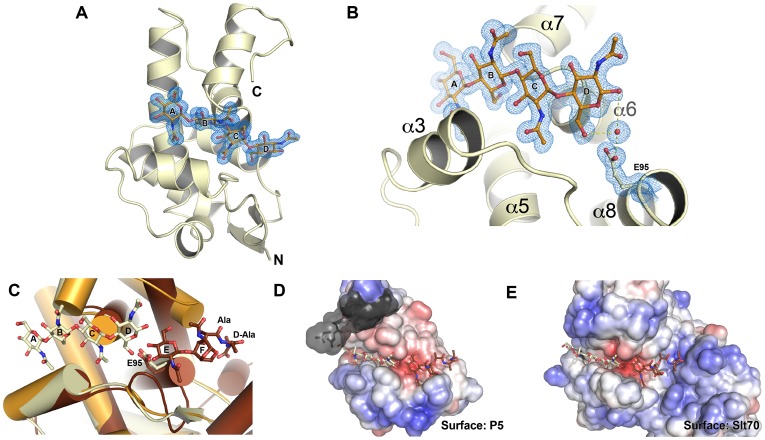
The structure of P5 bound to a glycan suggests lytic transglycosylase activity. A) Overall structure of P5ΔV8^wt^ bound to chitotetraose. The 2F_o_ - F_c_ electron density map for the ligand is shown contoured at 1 σ. Residues 199–220 are disordered. Helix α7 and the following linker are in slightly different positions than in the unliganded structure (see also Figure S3). The four NAG residues of chitotetraose bind to subsites A–D. B) Close-up of the active site. A water molecule is observed between Glu95 and the NAG in subsite D, supporting a lytic transglycosylase (LT) activity, with Glu95 as the catalytic acid/base. C) Superposition of ligand-bound P5ΔV8^wt^ (yellow) and apo-P5ΔV8^wt^ (orange) onto the structure of the *E. coli* slt70 LT (brown) containing a glycan product in subsites E and F (PDB 1QTD). The geometry and electrostatics of the P5 substrate-binding surface (D) are similar to those of slt70 (E). P5 residues displaced by the chitotetraose ligand are shown in grey with a semi-transparent surface.

The structure of Φ6 P5 bears the closest resemblance to G-type lysozymes and lytic transglycosylases (LTs) such as gp144 of phage ΦKZ [13] and the catalytic domain of *E. coli* slt70 with rmsds of 1.2 Å and 1.6 Å, respectively (Figure S3A–S3C). Like G-type lysozymes and LTs [13], [21], [22], [23], Φ6 P5 has just one glutamate, Glu95, in its predicted active site. To gain additional insight into the enzymatic activity of P5, we determined the structure of P5ΔV8^wt^ in complex with the substrate analog chitotetraose (NAG_4_), at 1.23 Å resolution. Chitotetraose is the most similar commercially available ligand to the natural substrates of LTs and ΦKZ gp144 binds chitotetraose [13]. The structure of ligand-bound P5ΔV8 is similar to that of the apo-enzyme with a few exceptions (Figure 4A, Figure S3G). The major difference is that residues 199–220 are missing from the ligand-bound structure. This region spans the last α-helix (α8) and includes the site of the selected V207F mutation. Helix α7 and the following linker are also in slightly different positions in the ligand-bound structure (Figure S3G). Additionally, the ligand displaces the side chain of Tyr196 out of the substrate-binding site, where the side chain is located in the apo-P5 structure (Figure 4). The ligand-bound P5 crystals belonged to a different space group with different crystal packing than the apo-P5 crystals. The substrate binding cleft is solvent-exposed in the ligand-bound crystals but mostly buried by non-crystallographic symmetry contacts in the apo-P5 crystals. Because the crystals of ligand-bound P5 took twice as long to grow as the apo-P5 crystals, we speculate that the lack of electron density for residues 199–220 in the ligand-bound structure is due to proteolytic cleavage by residual V8 protease or another contaminating protease in the P5 preparation. This cleavage may be favored by the increased solvent exposure of the C-terminal region in the ligand-bound crystals.

The substrate-binding sites of LTs and lysozymes have six sugar binding subsites (A–F) and substrate cleavage occurs between an N-acetyl muramic acid (NAM) bound in subsite D and a N-acetyl glucosamine (NAG) bound in subsite E [24], [25], [26]. In the ligand-bound P5 structure, the four NAG residues of the ligand bind to subsites A–D (Figure 4A). A water molecule is observed between Glu95 and the NAG in subsite D (Figure 4B), supporting a role of Glu95 as the acid/base in the catalytic mechanism. The ligand binds in the same manner as the identical chitotetraose ligand in the structure of the ΦKZ gp144 LT [13]. In both the P5 and gp144 structures, the mode of chitotetraose binding is consistent with the presence of the additional lactic acid and peptidyl moieties that are present in the NAM residues of natural substrates at subsites B and D. Moreover, a superposition of ligand-bound P5 onto the structure of *E. coli* slt70 LT bound to a glycan product in subsites E and F shows that the geometry and electrostatics of the P5 surface should allow binding of such a product in the same manner (Figure 4C, 4D). Together, the structural features of the P5 active site and glycan binding site and their similarity to the ΦKZ and slt70 LTs strongly support that P5 is a lytic transglycosylase, as previously predicted [17].

### P5^wt^ and P5^V207F^ have the same cell lysis activity

**Figure 5:**
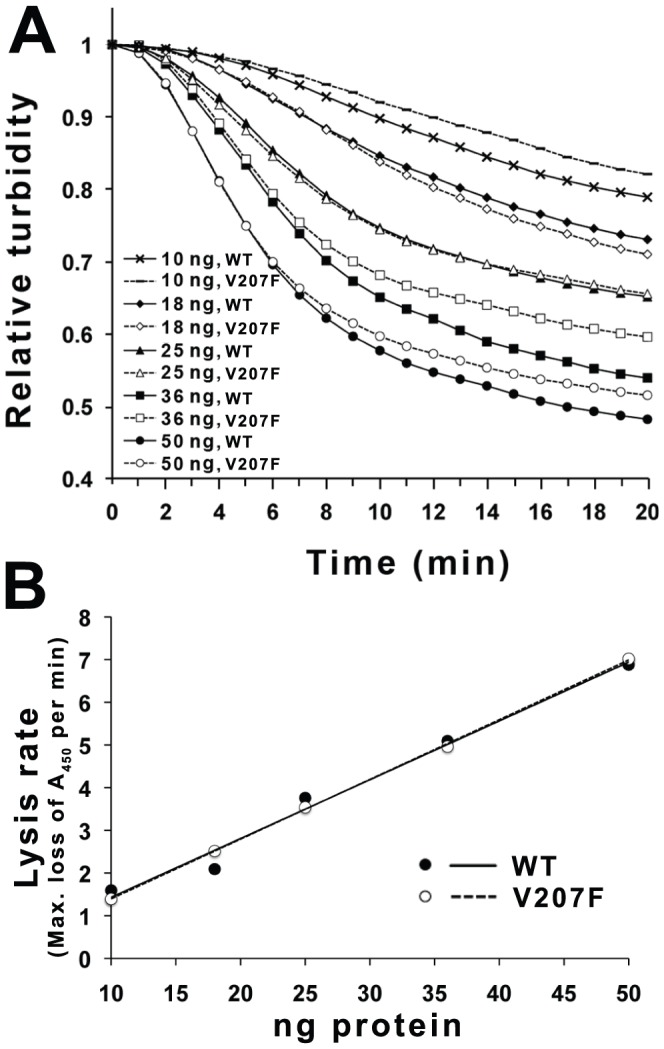
Cell wall lysis activity of P5^wt^ and P5^V207F^. A) To measure the cell lysis activities of P5^wt^ and P5^V207F^ the turbidity of chloroform-treated *E. coli* was measured as absorbance at 450 nm at 25°C. P5^wt^ and P5^V207F^ have the same maximum cell lysis rates. B) Linear relationship between the maximal rate of decrease in absorbance and the enzyme concentration.

To confirm that Φ6 P5 could lyse bacterial cell walls, we examined the cell lysis activity of P5^wt^ and P5^V207F^ using a turbidity assay at the normal growth temperature (25°C). Both proteins lysed cells with the same efficiency (Figure 5A). The maximal rates of cell lysis of P5^wt^ and P5^V207F^ were similar and were directly proportional to the enzyme concentration (Figure 5B).

## Discussion

Merging experimental evolution and structural biology, we used RNA phage Φ6 as a model to demonstrate how a single antagonistically pleiotropic mutation caused a survival/reproduction tradeoff in evolving populations. The V207F mutation conferred better survival of viruses under 50°C heat-shock, despite reducing their reproduction at 25°C; these data demonstrated that thermotolerance was the most important fitness component for dictating the overall evolutionary success of the treatment populations.

We are aware of few other studies that have examined survival/reproduction tradeoffs in viruses. de Paepe and Taddei compared lytic phages of *E. coli* and suggested a survival/reproduction tradeoff mediated by capsid structure [27]. The proposed mechanism was that denser-packaging of viral DNA within capsids affords increased stability, but tends to slow the rate of phage genome replication. Moreover, in experimental evolution studies where *E. coli* bacteria and phage ΦX174 or the related phage ID11 were exposed to elevated temperatures, results showed that changes in viral capsid proteins were likely stabilizing [28], [29]. Our results differed from these previous studies because we found the tradeoff was governed by an enzyme, rather than changes to the phage capsid. Also, we observed strong convergence evidenced by a single mutation that fixed in the independently evolved treatment populations, whereas the experiment with phage ID11 showed multiple possible first-step substitutions [28], [29]. One possibility is that our heat-shock regime selected strongly for structural stability, a single fitness component, whereas the phage ID11 study required growth of phage and bacteria at high temperature, thus selecting on multiple fitness components allowing various beneficial mutations to fix. Apparently, our selective regime was so stringent that the convergent mutation fixed despite strong antagonism for growth at ordinary temperature, which constituted environmental conditions aside from the 5 min heat shock. Further research could explore how this tradeoff may be lessened (or even eliminated) via further molecular change(s) resulting from fixation of mutations that compensate for the growth deficit.

A previous report suggested that the Φ6 P5 lysin might have endopeptidase activity rather than the glycanhydrolase activity of lysozyme [16]. However, the cell lysis activity assay used in that study cannot distinguish between cell wall lysis due to endopeptidase activity from cell wall lysis due to glycosidase activity. Conversely, a bioinformatics study by Pei and Grishin classified Φ6 P5 as a distant relative of the lytic transglycosylase (LT) subfamily within the lysozyme superfamily [17]. LTs are enzymes that can degrade the peptidoglycan layer of the bacterial cell wall by cleaving a β(1,4)-glycosidic bond between NAM and NAG residues and forming a new glycosidic bond between the O6 and C1 atoms of the NAM residue [30], [31]. In general, lysozymes possess a catalytic dyad of glutamic and aspartic acid residues that catalyze the hydrolysis of the same substrate by using a water molecule from the solvent [32], [33], [34], [35]. However, in LTs, there is only one acidic residue, typically a glutamic acid, in the vicinity of the substrate cleavage site [36]. Φ6 P5 adopts a lysozyme superfamily fold and our structure of P5 bound to a tetrasaccharide substrate analog shows that the enzyme contains a single glutamate (Glu95) in its active site. Together, our structural data unambiguously identify Φ6 P5 as an LT.

The lysozyme superfamily has been extensively studied as a model system for protein folding and stability, as reviewed in ref. [37]. Several hydrophobic cavities have been identified in lysozymes and it has been proposed that mutations filling these cavities should stabilize the protein by increasing the hydrophobic surface area buried within the fold [38]. However, attempts to fill two different hydrophobic cavities in T4 lysozyme by site-directed mutagenesis resulted unexpectedly in a slight decrease in the melting point of the protein, because stabilization from the increased hydrophobic contacts was offset by strain within the mutant side chains [38]. In contrast, the naturally selected V207F mutation in Φ6 P5 achieves the stabilization expected from the addition of four carbon atoms with a surface area of 35–40 Å^2^, ∼1 kcal/mol (or a 4°C increase in melting temperature), without the introduction of strain within the protein. Consistent with the average increase in the melting temperature of P5^V207F^ of 5.7°C reported here, binding of benzene to the cavity created by the L99A substitution in T4 lysozyme resulted in a 6.0°C increase in the melting temperature of T4 lysozyme [20]. We conclude that the enhanced thermal stability of P5^V207F^ is responsible for the survival of the mutant phages after the heat-shock challenge.

The V207F mutation did not affect the structure of the P5 active site, allowing the mutant enzyme to fully maintain its functional role as a lysin, which is essential in the viral lifecycle [12], [13]. Thus, it is not clear why the mutation adversely affected viral reproduction. We speculate that the mutation may reduce the structural plasticity of P5, and may hence reduce the efficiency of the assembly or disassembly of the viral capsid. Indeed, the architecture of quasiequivalent icosahedral viral capsids, such as that of Φ6, necessarily depends on structural plasticity within the capsid proteins to form the contacts that hold the capsid together in multiple nonequivalent environments. In support of this hypothesis, an electron cryomicroscopy structure of phage Φ12 (a cystovirus closely related to Φ6) at 10 Å resolution suggests that P5 in an integral part of the viral capsid [14]. Moreover, the weak electron density for P5 in the Φ12 structure suggests that P5 has some flexibility relative to the rest of the capsid, and this flexibility of P5 has been proposed to allow other proteins to access the capsid during virus replication [14]. The selected V207F mutation may therefore impair viral replication, and hence reproduction, by reducing the flexibility of P5.

Although it is widely recognized that infectious viruses can differ markedly in terms of their stability in the face of environmental stress, the associated effects of individual viral proteins remain largely unexplored. Medically and agriculturally important viruses sometimes show an inherent tendency to survive for extended periods outside of their hosts, suggesting that survivability should factor heavily in the relative transmission success of virus genotypes. This is likely to be true for variants of viruses that are transmitted between hosts via inert objects such as transmission of Hepatitis C Viruses between injection-drug users that share needle syringes [39]; better studied examples include differing ability of Influenza A Virus genotypes to withstand exposure to cold water when undergoing fecal-oral transmission in avian hosts [40]. Fever is generally assumed to be a useful innate defense against infecting viruses because these pathogens can degrade when exposed to elevated temperature; although this assumption is critical to the current debate of whether fever-reducing drugs ultimately harm or benefit infected hosts, it is perhaps surprising that evolution of temperature tolerance in viruses is seldom studied [28], [29], [41]. Our study indicated that evolved thermotolerance is rapidly acquired in RNA viruses selected under temperatures much higher than those they normally encounter, strongly suggesting that simple solutions (i.e., point mutations) may govern this adaptation in other virus systems.

## Materials and Methods

### Strains and culture conditions

Cultures of *Pseudomonas syringae* pathovar *phaseolicola* (ATCC #21781) host bacteria were initiated by a single colony grown at 25°C in LC medium: Luria-Bertani broth at pH 7.5. Phage were grown by mixing ∼100 particles with 200 µl of overnight bacterial culture in 3 ml 0.7% LC top agar, overlaid on a 1.5% LC agar plate. After 24 h, phage lysates were prepared by harvesting viral plaques into LC broth, followed by centrifugation and filtration to remove bacteria. Viral stocks were stored at −20°C in 2∶3 glycerol/LC (v/v). Bacterial stocks were stored in 2∶3 glycerol/LC(v/v) at −80°C.

### Experimental evolution

Clones of wildtype Φ6 (strain #PT522) were used to found three treatment and three control populations. Treatment populations were incubated in the absence of cells for 5 min at 50°C followed by 24 h of growth (5 virus generations) on a lawn of *P. phaseolicola* at 25°C. Viral progeny were harvested as described above. This process was repeated for a total of 20 passages (100 generations [42]) while monitoring the bottleneck population size of evolving lineages to ensure that they experienced equal generation numbers. Control populations were maintained identically but experienced periodic mock heat shocks at 25°C.

### Paired-growth and survival assays

Relative reproduction of virus strains was estimated in paired-growth assays as described [43]. Reproduction was gauged relative to common competitor phage: wildtype phage Φ6 containing an engineered mutation (fragment of the *Escherichia coli lacZ* gene for beta-galactosidase) on segment L [15]. We mixed the test phage and marked competitor at a 1∶1 volumetric ratio, and then plated a dilution of this mixture containing ∼200 viruses onto a host lawn of bacteria. After 24 h incubation, the resulting plaques were harvested and filtered to obtain a cell free lysate. We tracked the ratio of test virus to marked competitor in the starting mixture (*R*_0_) and in the harvested lysate (*R*_1_) by plating on lawns of LM1034: *P. phaseolicola* containing the complementing fragment of the *E. coli lacZ* gene. LM1034 allows the marked competitor to produce blue plaques on agar containing X-gal (0.4% w/v), whereas unmarked phage produce colorless plaques. We defined reproductive fitness (*W*) as the relative change in ratios, *W = R*_1_ */R*_0_. After log-transforming fitness estimates, mean log fitness of the wildtype strain was calculated and this value was subtracted from all fitness estimates to adjust for cost of the genetic marker on the common competitor. Fitness assays conducted under the treatment conditions used in experimental evolution (Figure S1) incorporated 5 min heat shock at 50°C. Plating a sample of the starting mixture onto a bacterial lawn confirmed the 1∶1 initial ratio (*R*_0_). An additional sample of the mixture was subjected to 5 min heat shock at 50°C, followed by plating on a lawn for plaque growth at 25°C; resulting plaques were harvested and titered to estimate the final ratio (*R*_1_). These data were analyzed as above, to estimate fitness in the treatment environment.

Survival was assayed as described [15]; 120 µl of a virus lysate containing ∼10^8^ particles was diluted onto a *P. phaseolicola* lawn to confirm the initial virus titer (*N*_i_). The lysate was then heated for 5 min and the final titer (*N*_f_) was measured. Percent survival equaled (*N*_f_/*N*_i_) * 100. Thus, survival under heat shock was gauged by tracking pfu viable for growth at 25°C.

### Sequencing

Genomic RNA was extracted (QiaAMP viral RNA extraction kit; Qiagen) and converted to cDNA by RT-PCR with Superscript polymerase and random hexamer primers (Invitrogen). Standard PCR methods were used to amplify 93.2% of the genome excluding the single-stranded ends of each segment [2]. PCR products were purified for sequencing with ExoSAP-It (US Biological). Virus genomes were sequenced with double coverage of every nucleotide. Sequences were analyzed with CLC DNA Workbench 6 (www.clcbio.com).

### Protein expression and purification

Genes encoding P5^wt^ and P5^V207F^ were cloned into the pET-28 vector (Novagen) in frame with an N-terminal six-histidine tag followed by a tobacco etch virus (TEV) protease cleavage site. P5^wt^ and P5^V207F^ were expressed in *E. coli* Rosetta (DE3) cells and (Novagen) purified by nickel-affinity and size-exclusion chromatography. The histidine tag was removed with 1∶100 (w/w) TEV protease (12 h at 16°C). Uncleaved P5 and TEV protease were removed with nickel-agarose beads. The proteins were stored at −80°C in 10 mM Tris pH 8, 0.1 M NaCl. P5ΔV8^wt^ and P5ΔV8^V207F^ were prepared by treating purified P5^wt^ and P5^V207F^ with 200∶1 (w/w) *S. aureus* V8 protease (Worthington) for 3 h. The protease was inactivated with 3 mM PMSF and Complete protease inhibitors (Roche). The P5ΔV8 proteins were then purified by size-exclusion chromatography.

### Limited proteolysis

V8 (Glu-C) protease (Worthington) was added to 0.3 g/l of P5^wt^ or P5^V207F^ to a molar ratio of 200∶1 P5∶V8, incubated on ice for 0.5–18 h and heat inactivated (95°C, 5 min in SDS-PAGE loading buffer). Proteolytic products were purified by reverse phase chromatography with a C4 column (Vydac) in 0.05% trifluoroacetic acid using a 10–80% acetonitrile gradient. Peak elution fractions were analyzed by MALDI mass spectroscopy at the Yale Chemical Instrumentation Center.

### CD measurements and thermal melting curves

Circular dichroism measurements were performed on an Aviv 202 spectrometer using 1mm path length cell. Protein samples were diluted to 0.3 g/l in 5 mM sodium phosphate pH 8 to give a reading of approximately −30 millidegrees at 220 nm. For melting curves the temperature was increased from 4°C to 95°C in 1° increments. Readings were taken every degree and were averaged over 3 s after 3 min of temperature equilibration.

Spectra were measured between 180 nm and 260 nm at scan rate of 1 nm/s. P5^wt^ and P5ΔV8^wt^ concentration was 5.5 µM and 4 µM, respectively. The raw data were corrected by subtracting the contribution of the buffer to CD signal. Data were smoothed and converted to molar ellipticity. The measurements were taken at a constant temperature of 16°C. The signal of residues 1–47 was calculated by subtracting the signal of P5ΔV8^wt^ from that of P5^wt^ after correcting for concentration and number of amino acid residues in terms of molar ellipticity.

For differential scanning calorimetry, P5 proteins were diluted to 20 µM in 10 mM Tris pH 8, 0.2 M NaCl and subjected to thermal scans from 10°C to 100°C at a rate of 60°C/h in a MicroCal VP calorimeter with a 15 min pre-equilibration time. Protein-free buffer was used as the reference. Data were collected in triplicate and analyzed with Origin 7 (OriginLab).

### Crystallization and structure determination of P5ΔV8

Crystals were grown by vapor diffusion at 16°C. P5ΔV8 at 7 g/l in 10 mM Tris pH 8, 0.1 M NaCl was mixed with a half-volume of reservoir solution (1.6 M sodium acetate, 0.1 M sodium citrate pH 6.5). Crystals were frozen in mother liquor. For the ligand-bound structure, P5ΔV8^wt^ was co-crystallized with a 10-fold molar excess of chitotetraose (Sigma) added 3 h prior to mixing with a half-volume of reservoir solution (15% PEG 3350, 0.2 M KNO_3_ pH 6.9). Two rounds of streak seeding into pre-equilibrated drops of reservoir solution were required to obtain single ligand-bound crystals, which were frozen in reservoir solution plus 25% (v/v) glycerol. P5ΔV8^wt^ crystals were derivatized by soaking in reservoir solution plus 0.2 M NaI for 45 s followed by freezing at 100 K. The structure was determined by single-wavelength anomalous diffraction with HKL2MAP [44]. The atomic model was built with ARP/wARP [45] and refined with Coot [46] and PHENIX [47]. The structure of ligand-bound P5ΔV8^wt^ was determined by molecular replacement with PHENIX using P5ΔV8^wt^ as the search model. Cavities were identified and analyzed with VOIDOO [48] using a 1.1 Å probe radius. See Table S2 for data collection and refinement statistics. Atomic coordinates and structure factors for P5ΔV8^wt^, P5ΔV8^V207F^ and ligand-bound P5ΔV8^wt^ were deposited in the Protein Data Bank (ID codes 4DQ5, 4DQ7 and 4DQJ).

### Cell lysis activity assay

To assay the cell lysis activities of P5^wt^ and P5^V207F^, the decrease in turbidity of a chloroform-treated *E. coli* culture was measured as described [16] by tracking absorbance at 450 nm and 25°C for 20 min after addition of 10–50 ng of protein. For additional details, see Extended Materials and Methods (Text S1).

## Supporting Information

Figure S1 Design for experimental evolution of phage Φ6 populations, passaged in the presence and absence of 50°C heat shock. (A) Wildtype phage Φ6 was plated on a lawn of *Pseudomonas syringae* pathovar *phaseolicola* bacteria, and three plaques were chosen at random to found a pair of ‘sister’ lineages. Treatment lineages (T1 thru T3) were passaged in the presence of periodic 50°C heat shock, whereas Control lineages (C1 thru C3) experienced mock heat shock of 25°C. (B) For experimental passage, each lineage experienced the survival assay at 50°C, followed by sampling to create a dilution series on host lawns. After overnight incubation at 25°C, the dilution yielding ∼10^3^ pfu was harvested and filtered to obtain a new cell-free lysate. The survival assay and plating were then repeated using naïve (non-coevolved) bacteria. This propagation scheme was repeated for 20 consecutive days (100 generations of phage evolution) where heat shock occurred every fifth generation.
(PDF)

Figure S2 The N-terminus of P5 lacks secondary structure. The CD spectrum of P5^wt^ is shown in blue, the CD spectrum of P5^wt^ΔV8 is in red, and the difference CD signal between them representing the signal coming from residues 1–47 is in black. The minimum at 190 nm is indicative of random coil conformation. Data were corrected for sample concentration and number of amino acid residues.
(PDF)

Figure S3 Comparison of the P5ΔV8^wt^, P5ΔV8^V207F^ and ligand-bound P5ΔV8^wt^ structures to each other and to other members of the lysozyme superfamily. (A)–(E) Superposition of the P5ΔV8^wt^ structure onto the structures of representative members of the lysozyme superfamily: A) 3BKV (catalytic domain only); B) 3GRX; C) 1QTD (catalytic domain only); D) 2Y8P; E) 148L. F) The P5ΔV8^wt^ and P5ΔV8^V207F^ crystals have unusual crystal packing. Three residues at the N-terminus of one of the two subunits in the asymmetric unit (blue) interact with the C-terminal lobe of a subunit in an adjacent asymmetric unit (white). G) Superposition of P5ΔV8^wt^ (blue), P5ΔV8^V207F^ (red) and chitotetraose-bound P5Δ8^wt^ (orange). In the chitotetraose -bound structure, residues 48–60 and 199–220 are disordered and residues 195–198 adopt a different orientation than in the unliganded structures. The three structures have essentially identical backbone conformations in residues 61–194, including around the active site.
(TIF)

Figure S4 Structural detail of the hydrophobic cavity filled by the selected V207F mutation. Left panel: within the core of the P5Δ8^wt^ structure (cyan), an unoccupied cavity with a volume of 30.28 Å^3^ (green mesh) is located near residue 207. Right panel: close-up showing the area boxed in the left panel. In the selected V207F mutant, the phenylalanine side chain (orange) fills the cavity (green mesh). The cavity surface was calculated using 1.1 Å probe radius.
(TIF)

Table S1 Mutations observed in the consensus sequencing of the evolved treatment and control phage populations.
(DOC)

Table S2 Crystallographic data collection and refinement statistics.
(DOC)

Text S1 Extended Materials and Methods and Supplementary References.
(DOC)
