# Correction: Selective Pressure Causes an RNA Virus to Trade Reproductive Fitness for Increased Structural and Thermal Stability of a Viral Enzyme

**DOI:** 10.1371/annotation/aa9bff6f-92c4-4efb-9b7f-de96e405e9d3

**Published:** 2012-12-12

**Authors:** Moshe Dessau, Daniel Goldhill, Robert C. McBride, Paul E. Turner, Yorgo Modis

The third author’s name was spelled incorrectly. The correct name is: Robert C. McBride. The correct citation is: Dessau M, Goldhill D, McBride RC, Turner PE, Modis Y (2012) Selective Pressure Causes an RNA Virus to Trade Reproductive Fitness for Increased Structural and Thermal Stability of a Viral Enzyme. PLoS Genet 8(11): e1003102. doi:10.1371/journal.pgen.1003102

