# Supplementary material for: Selective Pressure Causes an RNA Virus to Trade Reproductive Fitness for Increased Structural and Thermal Stability of a Viral Enzyme
Source: PLoS Genet. 2012 Nov 29;8(11):e1003102. doi: 10.1371/journal.pgen.1003102 (PMC3510033; doi:10.1371/journal.pgen.1003102)
Supplement: Figure S1 — Design for experimental evolution of phage Φ6 populations, passaged in the presence and absence of 50°C heat shock. (A) Wildtype phage Φ6 was plated on a lawn of Pseudomonas syringae pathovar phaseolicola bacteria, and three plaques were chosen at random to found a pair of ‘sister’ lineages. Treatment lineages (T1 thru T3) were passaged in the presence of periodic 50°C heat shock, whereas Control lineages (C1 thru C3) experienced mock heat shock of 25°C. (B) For experimental passage, each lineage experienced the survival assay at 50°C, followed by sampling to create a dilution series on host lawns. After overnight incubation at 25°C, the dilution yielding ∼103 pfu was harvested and filtered to obtain a new cell-free lysate. The survival assay and plating were then repeated using naïve (non-coevolved) bacteria. This propagation scheme was repeated for 20 consecutive days (100 generations of phage evolution) where heat shock occurred every fifth generation. (PDF) [file pgen.1003102.s001.pdf]

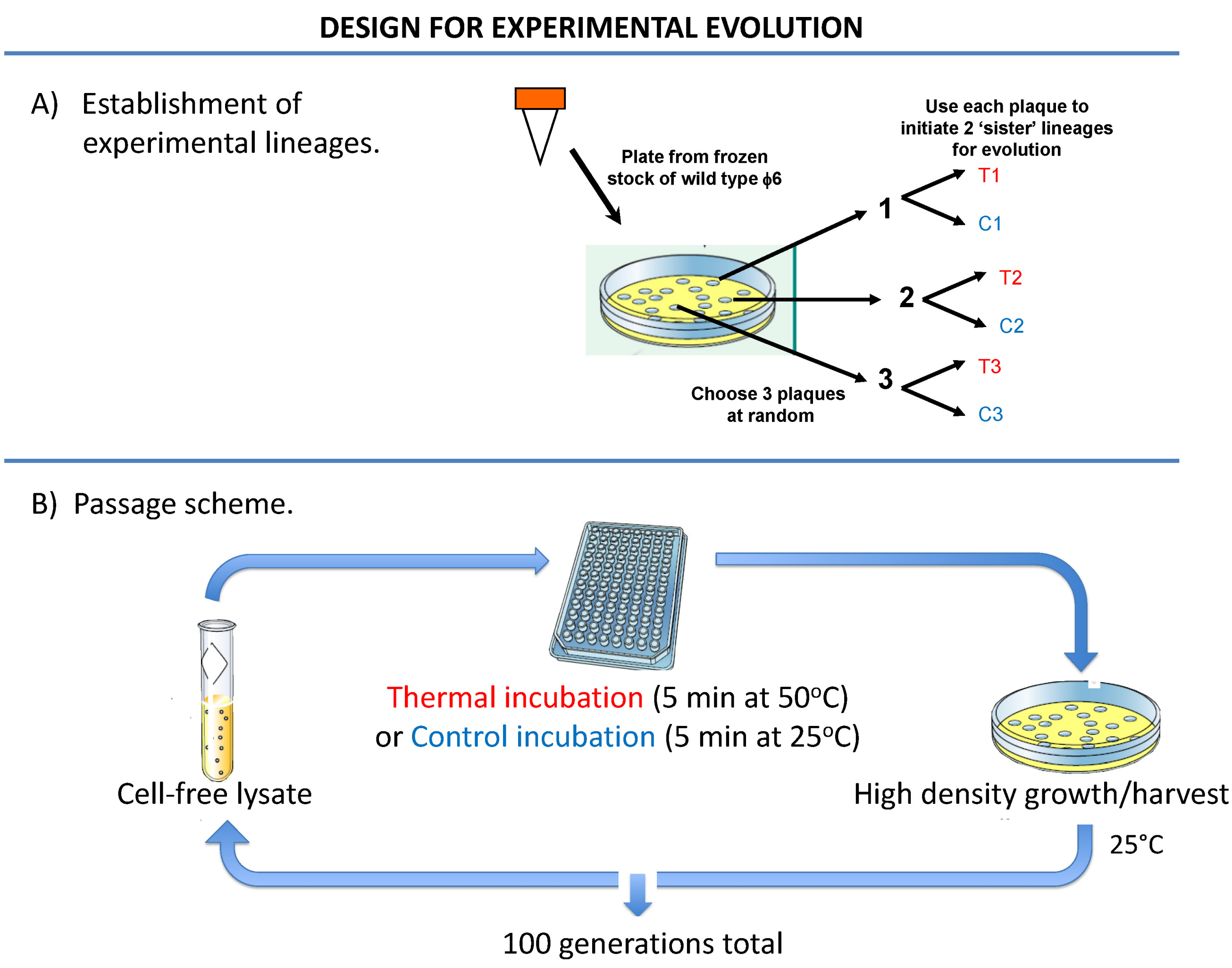

Supplementary Figure 1
