# Supplementary figures and images for: Selective Pressure Causes an RNA Virus to Trade Reproductive Fitness for Increased Structural and Thermal Stability of a Viral Enzyme
Source: PLoS Genet. 2012 Nov 29;8(11):e1003102. doi: 10.1371/journal.pgen.1003102 (PMC3510033; doi:10.1371/journal.pgen.1003102)

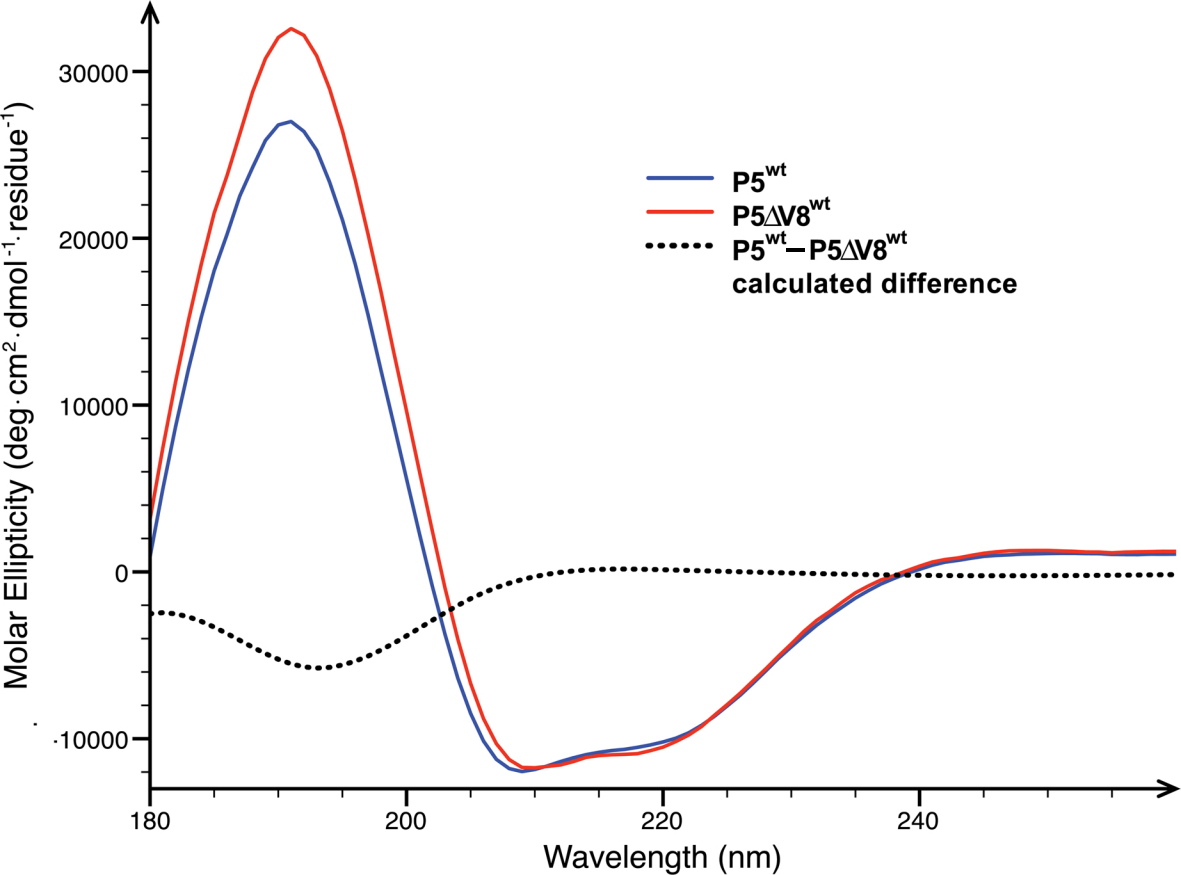

**Supplementary Figure 2**

Supplement: Figure S2 — The N-terminus of P5 lacks secondary structure. The CD spectrum of P5wt is shown in blue, the CD spectrum of P5wtΔV8 is in red, and the difference CD signal between them representing the signal coming from residues 1–47 is in black. The minimum at 190 nm is indicative of random coil conformation. Data were corrected for sample concentration and number of amino acid residues. (PDF) [file pgen.1003102.s002.pdf]

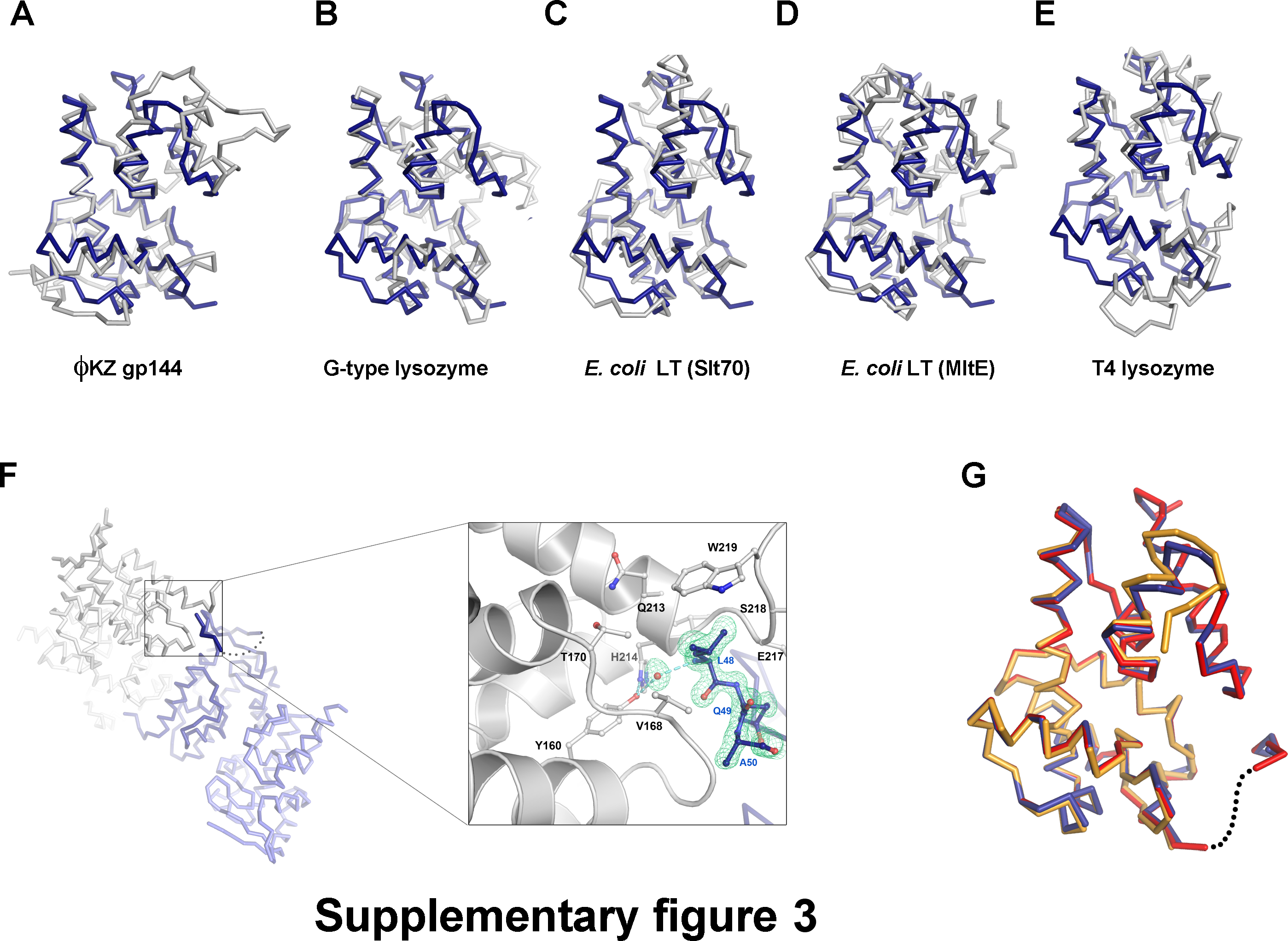

Supplement: Figure S3 — Comparison of the P5ΔV8wt, P5ΔV8V207F and ligand-bound P5ΔV8wt structures to each other and to other members of the lysozyme superfamily. (A)–(E) Superposition of the P5ΔV8wt structure onto the structures of representative members of the lysozyme superfamily: A) 3BKV (catalytic domain only); B) 3GRX; C) 1QTD (catalytic domain only); D) 2Y8P; E) 148L. F) The P5ΔV8wt and P5ΔV8V207F crystals have unusual crystal packing. Three residues at the N-terminus of one of the two subunits in the asymmetric unit (blue) interact with the C-terminal lobe of a subunit in an adjacent asymmetric unit (white). G) Superposition of P5ΔV8wt (blue), P5ΔV8V207F (red) and chitotetraose-bound P5Δ8wt (orange). In the chitotetraose -bound structure, residues 48–60 and 199–220 are disordered and residues 195–198 adopt a different orientation than in the unliganded structures. The three structures have essentially identical backbone conformations in residues 61–194, including around the active site. (TIF) [file pgen.1003102.s003.tif]

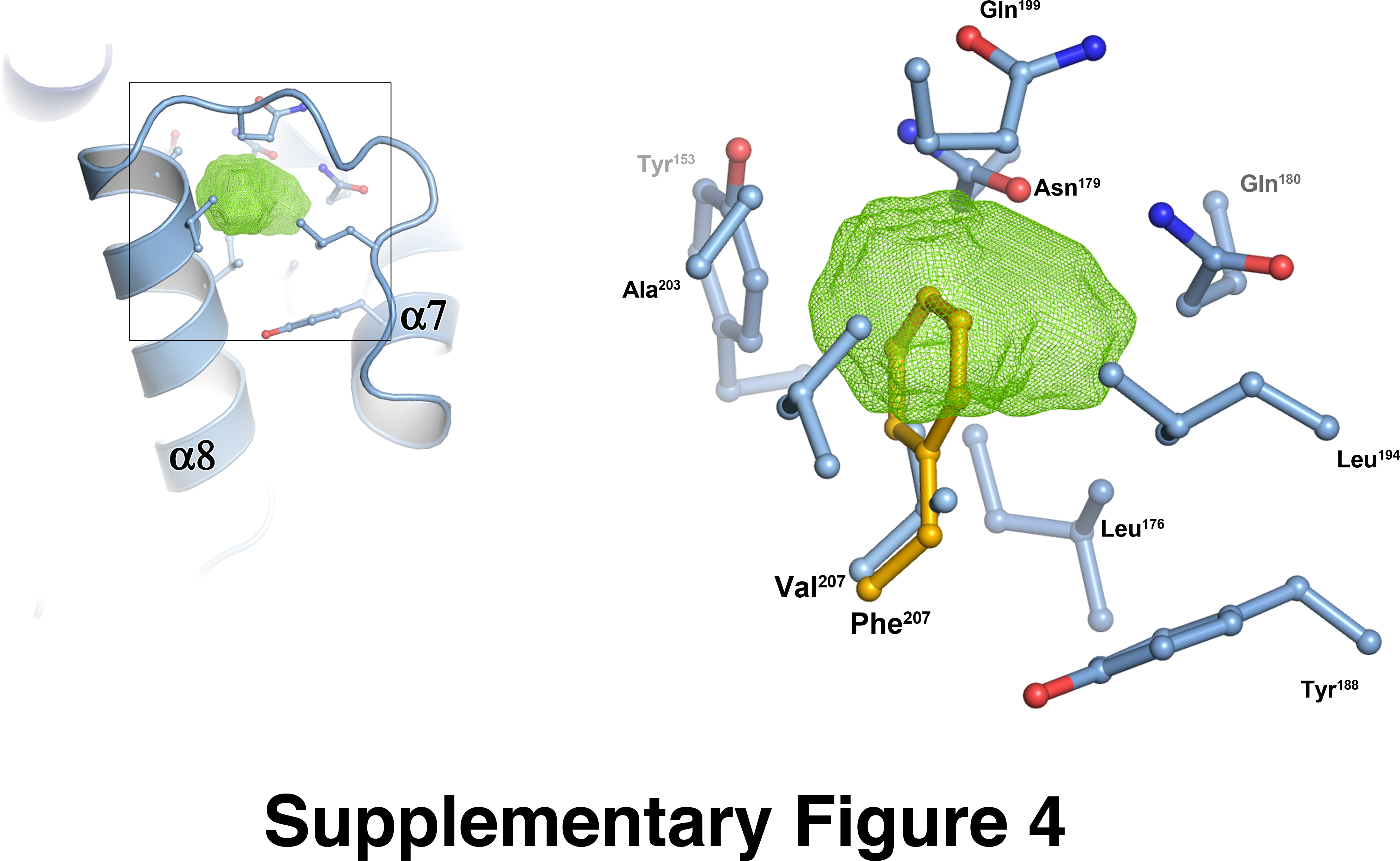

Supplement: Figure S4 — Structural detail of the hydrophobic cavity filled by the selected V207F mutation. Left panel: within the core of the P5Δ8wt structure (cyan), an unoccupied cavity with a volume of 30.28 Å3 (green mesh) is located near residue 207. Right panel: close-up showing the area boxed in the left panel. In the selected V207F mutant, the phenylalanine side chain (orange) fills the cavity (green mesh). The cavity surface was calculated using 1.1 Å probe radius. (TIF) [file pgen.1003102.s004.tif]
