# Supplementary material for: Selective Pressure Causes an RNA Virus to Trade Reproductive Fitness for Increased Structural and Thermal Stability of a Viral Enzyme
Source: PLoS Genet. 2012 Nov 29;8(11):e1003102. doi: 10.1371/journal.pgen.1003102 (PMC3510033; doi:10.1371/journal.pgen.1003102)
Supplement: Table S1 — Mutations observed in the consensus sequencing of the evolved treatment and control phage populations. (DOC) [file pgen.1003102.s005.doc]

Table S1. Mutations observed in the consensus sequencing of the evolved treatment and control phage populations.

| Base Pair | Small 2238 | Medium 491 | 492 | 877 | 2268 | 3554 | Large 657 | 767 | 4688 |
| --- | --- | --- | --- | --- | --- | --- | --- | --- | --- |
| Amino acid Change | V->F | K->T | K->N | No Change | No Change | No Change | V->A | M->L | No change |
| Base pair Change | G->T | A->C | G->C | T->C | G->A | G->A | T->C | A->C | G->A |
| Control 1 | G | C | G | T | G | G | T | A | G |
| Control 2 | G | C | G | T | G | G | T | A | G |
| Control 3 | G | A | C | T | G | A | T | A | G |
| Treatment 1 | T | A | G | C | A | G | C | A | G |
| Treatment 2 | T | A | C | T | A | A | T | C | A |
| Treatment 3 | T | A | G | C | A | G | C | A | G |
