# Supplementary material for: Selective Pressure Causes an RNA Virus to Trade Reproductive Fitness for Increased Structural and Thermal Stability of a Viral Enzyme
Source: PLoS Genet. 2012 Nov 29;8(11):e1003102. doi: 10.1371/journal.pgen.1003102 (PMC3510033; doi:10.1371/journal.pgen.1003102)
Supplement: Table S2 — Crystallographic data collection and refinement statistics. (DOC) [file pgen.1003102.s006.doc]

Table S2. Crystallographic data collection and refinement statistics.

|  | P5ΔV8^wt^ | P5ΔV8^V207F^ | P5ΔV8^wt^-NaI | P5ΔV8 ^ligand^ |
| --- | --- | --- | --- | --- |
| **Data collection** |  |  |  |  |
| Space group | P2_1_2_1_2_1_ | P2_1_2_1_2_1_ | P2_1_2_1_2_1_ | P2_1_ |
| Cell dimensions |  |  |  |  |
| *a*, *b*, *c* (Å) | 62.22, 68.92, 90.15 | 62.12, 68.72, 89.89 | 62.04, 68.43, 89.34 | 43.88, 50.10, 65.11 |
| *α*, *β*, *γ* (º) | 90, 90, 90 | 90, 90, 90 | 90, 90, 90 | 90, 103.43, 90 |
| Wavelength (Å) | 1.075 | 1.075 | 1.542 | 0.9792 |
| Resolution (Å)^a^ | 60-1.4 (1.42-1.40) | 55-1.4 (1.42-1.40) | 50-1.9 (1.93-1.90) | 50-1.2 (1.25-1.23) |
| Unique reflections | 77,074 | 73,833 | 49,720 | 77,799 |
| *R*_merge_ (%)^a,b^ | 5.8 (63.8) | 5.2 (98.0) | 9.4 (95.9) | 6.0 (99.8) |
| *I*/σ*I^a^* | 35 (1.45) | 37.5 (1.03) | 23.8 (1.12) | 27.1 (1.4) |
| Completeness (%)^a^ | 99.8 (100) | 96.5 (94.5) | 86 (32) | 95 (83.5) |
| Redundancy^a^ | 4.9 (4.1) | 4.3 (4.1) | 6.6 (3) | 3.4 (3.2) |
| **Refinement** |  |  |  |  |
| Resolution (Å) | 60-1.4 | 55-1.4 |  | 50-1.23 |
| No. reflections | 77,006 | 73,824 |  | 77,752 |
| *R*_work_, *R*_free_^c^ | 16.4, 17.7 | 17.5, 19.7 |  | 15.0, 16.9 |
| Average B factor (Å^2^)^b^ | 25.4 | 31.0 |  | 17.5 |
| R.M.S deviations |  |  |  |  |
| Bond lengths (Å) | 0.005 | 0.005 |  | 0.011 |
| Bond angles (º) | 1.08 | 1.00 |  | 1.8 |
| Ramachandran plot | |  |  |  |
| Residues in preferred regions | 301 (98.4%) | 308 (98.7%) |  | 263 (99.6%) |
| Residues in additional allowed regions | 5 (1.6%) | 4 (1.3%) |  | 1 (0.4%) |
| Residues in disallowed regions | 0 | 0 |  | 0 |

^a^Values for the highest resolution shell are shown in parentheses.

^b^R_merge_ = Σ_hkl_Σ_i_ |I_hkl,i_ – < I>_hkl_| / Σ_hkl_Σ_i_|I_hkl,i_|, where I_hkl_ is the intensity of a reflection and <I>_hkl_ is the average of all observations of the reflection.

^c^Residual B-factors after TLS refinement. See PDB entries for TLS refinement parameters.

^d^R.M.S., root mean square

^e^R_free_, R_work_ with 10% of Fobs sequestered before refinement.
