# Supplementary material for: Selective Pressure Causes an RNA Virus to Trade Reproductive Fitness for Increased Structural and Thermal Stability of a Viral Enzyme
Source: PLoS Genet. 2012 Nov 29;8(11):e1003102. doi: 10.1371/journal.pgen.1003102 (PMC3510033; doi:10.1371/journal.pgen.1003102)
Supplement: Text S1 — Extended Materials and Methods and Supplementary References. (DOC) [file pgen.1003102.s007.doc]

**EXTENDED MATERIALS AND METHODS**

**Protein expression and purification**

P5 expression in *E. coli* was induced with 0.5 mM IPTG at OD_600_ = 0.6 for 4 h. Cell were resuspended in Lysis buffer (50 mM sodium phosphate pH 8.0, 0.3 M NaCl, 0.1% Triton X-100, 10 g/l lysozyme, 1500 U DNase and 5% glycerol). P5 was partially purified from the cell lysate by nickel-affinity chromatography with Ni-NTA beads (QIAgen) in Buffer A (50 mM sodium phosphate pH 8.0, 300 mM NaCl and 5% glycerol). P5 was then incubated with TEV protease (1:100 w/w) for 12 h at 16˚C while dialyzing against Buffer A to remove the imidazole from the nickel elution step. Uncleaved P5 and TEV protease were removed with Ni-NTA beads and P5 was further purified by size exclusion chromatography on Superdex 200 10/300 GL column (GE Healthcare) in 10 mM Tris pH 8.0, 0.1 M NaCl. Concentrated samples were stored at −80˚C.

**Crystallization and structure determination of P5∆V8^wt^, P5∆V8^V207F^ and ligand-bound P5∆V8^wt^**

P5ΔV8 crystals appeared after 5 h and were frozen in mother liquor 3-7 days after setup with sizes ranging from 0.15 to 0.3 mm in the longest dimension. Crystals grew as clusters of thin needles. Two rounds of streak seeding into pre-equilibrated drops of reservoir solution were required to obtain single rod-shaped crystals longer than 200 µm, after 5-8 days.

Iodine sites were located and experimental phases were calculated with SHELX and HKL2MAP. Unliganded P5ΔV8 crystals belonged to space group P2_1_2_1_2_1_ with two molecules per asymmetric unit. ARP/wARP was used for automated model building. The model was improved with rounds of model building, refinement and water placement with Coot and REFMAC5 . Final refinement was performed with PHENIX.

**Cell lysis activity assay**

Aliquots of chloroform-treated *E. coli* were thawed and diluted with 950 µl of 50 mM Tris pH 8 with 0.1% Triton X-100. 10 – 50 ng of protein was diluted in 30 µl of 10 mM Tris pH 8 and then added to the bacteria. 30 µl of 10 mM Tris pH 8 without protein was added to bacteria and used as a blank. The change in absorbance was calculated at 1-min intervals and the maximum change was plotted against protein concentration (Figure 5).

**Supplementary References**

1. Sheldrick GM (2008) A short history of SHELX. Acta Crystallogr A 64: 112-22.

2. Murshudov GN, Vagin AA, Dodson EJ (1997) Refinement of macromolecular structures by the maximum-likelihood method. Acta Crystallogr D Biol Crystallogr 53: 240-55.
